# Effects of Intraperitoneal Colchicine on the Metabolic Consequences of High Fat Diet-Induced Obesity in Mice

**DOI:** 10.2174/0118715303415534251117044937

**Published:** 2026-02-04

**Authors:** Alexa P. Lyman, Jordan A. Levine, Brooke E. Arner, Blaise Stearns, Tushar P. Patel, Andrew P. Demidowich, Paul S. Blank, Joshua J. Zimmberberg, Jack A. Yanovski

**Affiliations:** 1 Section on Growth and Obesity, Division of Intramural Research (DIR), *Eunice Kennedy Shriver* National Institute of Child Health and Human Development (NICHD), National Institutes of Health (NIH), Bethesda, MD 20892, USA;; 2 Section on Integrative Biophysics, DIR, NICHD, NIH, Bethesda, MD20892, USA;; 3 Current Address: Johns Hopkins Community Physicians at Howard County Medical Center, Johns Hopkins Medicine, Columbia, MD, USA

**Keywords:** Colchicine, inflammation, drug toxicity, mouse, high-fat diet, insulin resistance

## Abstract

**Introduction:**

Obesity-associated inflammation promotes metabolic dysregulation and insulin resistance. Colchicine, a potent microtubule inhibitor, suppresses NLRP3 inflammasome activity and acts proximally to inhibit the inflammatory pathway. The primary objective was to evaluate the impact of intraperitoneal (IP) colchicine administration on inflammation and metabolic parameters in mice with high-fat diet-induced obesity.

**Methods:**

C57BL/6 mice consumed a 45% fat diet from age 8 weeks to 16 weeks to induce obesity and inflammation. From weeks 12-16, 39 male and 29 female mice were randomized 1:1 to IP vehicle or IP colchicine 0.2 mg/kg/d. Glucose and insulin tolerance tests were performed, and CRP was measured to evaluate systemic inflammation. NLRP3 and Caspase-1 expression were assessed to evaluate hepatic inflammation.

**Results:**

Body weight was lower for both male and female mice administered colchicine *versus* placebo (*p*<0.001). Serum CRP decreased in colchicine *vs* placebo in both male and female mice (*p*’s<0.05). However, there was no evidence to support a colchicine-induced change in hepatic NLRP3 and Caspase-1 expression. Despite attenuating weight gain, colchicine worsened whole-body insulin sensitivity during a post-treatment insulin tolerance test compared to placebo in male mice (*p* < 0.01).

**Discussion:**

In mice with high-fat diet obesity, IP colchicine 0.2 mg/kg for 4 weeks significantly suppressed inflammation but had no significant effect on markers of hepatic inflammasome activity. Colchicine treatment also induced considerable weight loss and worsened whole-body insulin sensitivity in male mice.

**Conclusion:**

Although this dose has been used in prior short-term mouse studies to suppress inflammation, chronic administration of 0.2 mg/kg intraperitoneal colchicine appears not suitable for metabolic studies.

## 1 INTRODUCTION

Numerous studies have shown that high-fat diets (HFDs) lead to an increase in fat mass and result in higher levels of circulating inflammatory molecules, including interleukin (IL)-1β, IL-6, and tumor necrosis factor-α (TNF-α) [1-5].

Mice fed a standard chow diet generally do not develop obesity and insulin resistance [6]. Adipose-derived inflammation is generally considered a crucial precursor to obesity-related comorbidities [7, 8].

Obesity-induced inflammation decreases insulin sensitivity in peripheral tissues. Inflammatory cytokines activate serine kinases [9, 10] that phosphorylate serine residues on membrane-bound IRS-1, deactivating IRS-1, and causing less Akt activity [10, 11], in turn reducing GLUT-4 plasma membrane translocation and reducing the cellular entry of glucose [12]. Cytokines also induce the suppression of cytokine signaling (SOCS) proteins, which would otherwise lead to reduced IRS-1. As a result, obesity leads to insulin-resistance and ultimately elevated blood glucose concentrations.

Systemic inflammation, even when low-grade, also disrupts beta cell function, reducing insulin secretion [13]. Such chronic inflammation causes mitochondrial stress, islet dysfunction, and eventual beta-cell apoptosis [12, 13]. This progression puts individuals with insulin resistance at risk for developing type 2 diabetes [14].

Mouse studies using anti-inflammatory agents have shown favorable effects on complications of obesity-related inflammation. Administration of an IL-1-beta antibody to mice on HFD improved glycemia [15], and prevented the onset of insulin resistance [16]. Similarly, administration of an IL-1 receptor blocker resulted in better beta-cell function in mice on an HFD [17]. However, obesity-induced inflammation is known to trigger a host of inflammatory pathways, and in human studies, interfering with a single pathway has not been sufficient to alter insulin sensitivity [18-20].

The inflammasome, referred to as “nod-like receptor family pyrin domain containing 3” (NLRP3), is a significant system promoting the inflammation caused by obesity [21]. Inhibiting the robust inflammatory process more proximally by targeting the assembly of the NLRP3 inflammasome may prove more effective at improving the downstream consequences of obesity-associated inflammation [7]. For example, among mice with dietary obesity, *Nlrp3^−/−^* mice (compared to controls) developed fewer pancreatic inflammatory consequences [22].

Importantly, microtubular function is critical for cytosolic localization and formation of the NLRP3 inflammasome [23, 24]. Specifically, danger-associated molecular patterns can increase alpha-tubulin acetylation [23], which in turn activates the dynein- and kinesin-1-dependent [25, 26] transport of mitochondrial-bound ASC [27] to localize with endoplasmic reticulum-bound NLRP3 and procaspase-1. This process also requires the actions of centrosomal kinase NIMA-related kinase 7 [28] and MARK4 [29], molecules involved in microtubular function. Once co-localized, the multiprotein assembly serves as a platform for caspase-1 activation, leading to the cleavage and activation of pro-inflammatory cytokines.

Colchicine is a potent inhibitor of microtubule polymerization and can proximally inhibit the NLRP3 inflammasome-induced cytokine storm by preventing the cytosolic localization of mitochondrial-bound ASC and endoplasmic-reticulum-bound NLRP3 [30]. When colchicine binds to beta-tubulin, it alters the normally straight tubulin structure of microtubules by introducing steric hindrance that disrupts the addition of alpha- / beta-tubulin heterodimers necessary for microtubular elongation [31] and by preventing heterodimer recycling [32], irreversibly affecting microtubular function.

There are somewhat conflicting data regarding the ability of colchicine to impact atherosclerosis-related disease by reducing inflammation [33-40]. However, recent meta-analyses have concluded that there is overall evidence of benefit [41, 42]. One pilot study in adults with obesity given colchicine or placebo for 3 months reported improvements in inflammatory secretome molecules, including C-reactive protein (CRP) [43], as well as greater fasting insulin sensitivity [44] in colchicine versus placebo treated individuals, marked immunomodulatory effects on peripheral blood mononuclear cell populations [45], and improvements in insulin regulation of lipolysis [46]. We hypothesized that, in high-fat diet-fed mice, intraperitoneal (IP) administration of colchicine would suppress obesity-linked inflammation through NLRP3 inflammasome inhibition and improve glucose homeostasis.

## 2 METHODS

### 2.1 Study Design

A prospective cohort study was conducted in mice, in which they were randomized to receive either a placebo or treatment with colchicine.

### 2.2 Animals

Starting at age 8 weeks, male (n=39) and female (n=29) C57BL/6 mice (Jackson Laboratory) were given for 8 weeks, a 45% HFD (45% kcal from fat, diet D12451, Research Diets, Inc., New Brunswick, NJ; https://researchdiets.com/formulas/d12451) ad lib, to induce obesity [47]. This sample size was expected to be sufficient based on prior studies using colchicine in mice [48-54]; therefore, no formal a priori power calculation was performed. Mice were on a 14-h light, 10-h dark cycle in the National Institutes of Health (NIH) vivarium. The experiment was performed in compliance with the NIH Animal Research Advisory Committee (ARAC) guidelines. Studies were approved as Animal Study Proposal 21.052 by the *Eunice Kennedy Shriver* National Institutes of Child Health and Human Development (NICHD) Animal Care and Use Committee (ACUC). There were no formal inclusion/exclusion criteria for experimental animals, and all data obtained are reported. There were no humane endpoints needed.

### 2.3 Treatment Randomization

After 4 weeks of HFD to induce baseline obesity and inflammation, the 12-week-old mice were randomly assigned to receive either 28 daily IP injections of placebo (normal saline) or 0.2 mg/kg colchicine. Investigators were not blinded to treatment. Body weight was measured every day throughout treatment. The daily intraperitoneal 0.2 mg/kg (20 µg/100 g) body weight (BW) colchicine dosage or equivalent volume of placebo was calculated per daily body weight with no maximum volume, but since mouse body weights were all below 40 g, the largest volume injected was <0.4 mL). This intraperitoneal dose of colchicine suppresses inflammation, and prior studies had suggested it was not associated with serious side effects in mice [53-55]. An experiment in 24 rats with diet-induced hyperlipidemia, which used an even higher daily colchicine dose of 0.5 mg/kg for 5 weeks [56], found that colchicine reduced inflammatory markers and endothelial dysfunction, but increased serum markers of hepatic damage (ALT and AST).

Colchicine USP powder (Spectrum Chemical, New Brunswick, NJ #CO115) was purchased and prepared in a sterile fashion in pharmaceutical-grade normal saline. 20 mg colchicine was dissolved in 10 mL of sterile saline to yield a stock concentration (2 mg/mL). The stock was filtered through a sterile 0.22-micron Milliex GP Filter. 20 microliter aliquots of stock were stored in sterile Eppendorf tubes (#C-3261-1) and frozen at -80°C. On the day of injection, stock aliquots were thawed, and 10 microliters of stock were dissolved in 990 microliters of normal saline (20 mcg/mL). The solution was refiltered through a 0.22-micron Milliex GP Filter under a sterile hood before being placed in syringes and administered to mice.

### 2.4 Body Composition

Mice underwent DEXA scans under anesthesia twice, at age 12 weeks (pre-randomization) and at 16 weeks (end-treatment). Each scan took approximately 4-5 minutes using the Lunar PIXImus apparatus (DEXA, Lunar, Madison, WI).

### 2.5 IP Glucose/Insulin Tolerance Tests (GTT, ITT)

At the end of treatment, as previously described [57], GTT (2 mg/kg BW IP dextrose diluted to 250 mg/mL, after an overnight 16 h fast) and ITT (0.75 U/kg BW IP insulin, after no food for 5 h) were done. For both GTT and ITT, serum glucose was measured using a glucometer as previously described [58], with 3-5 microliters of blood obtained from the tail vein after <1 cm of snipping every 30 minutes for 120 minutes.

### 2.6 Serum Inflammation Analysis

Serum CRP ELISA assays (Fisher Scientific #MCRP00) were performed using samples collected at age 16 weeks (end-treatment) to assess systemic inflammation. 125 μL serum was collected from the orbital sinus while the mice were anesthetized.

### 2.7 Hepatic Tissue Inflammation Proteins: Evaluation by Western Blot Assay

Liver samples were isolated post-mortem. Protein samples were homogenized (FastPrep-24), centrifuged, and isolated before long-term storage at -80°C. The samples were separated using 12% NuPAGE gels (Invitrogen, Carlsbad, CA). Primary antibodies for NLRP3 (AdipoGen #AG-20B-0014-C100, AdipoGen Corp, San Diego, CA) and Caspase-1 (AdipoGen #AG-20B-0042-C100), as previously described [59], were diluted to 1:1000 concentration and secondary antibodies were diluted to 1:2500 concentration. Cleaved (mature, ~22kD) Caspase-1 expression was determined. The values are presented relative to β-actin expression, measured using a primary Anti-β-actin antibody (Abcam # ab8229) diluted to a 1:500 concentration and a secondary antibody diluted to a 1:5000 concentration. Protein levels were quantified by image density scanning and analyzed using Empiria Studio (LI-COR Biotech, LLC., Lincoln, NE). The number of biological samples used for western blotting and analysis is listed in each individual graph.

### 2.8 Tissue Insulin Sensitivity Proteins: Evaluation By Western Blot Assay

Protein samples were separated using NuPAGE gels (Invitrogen, Carlsbad, CA). To examine insulin action, we evaluated expression of Serine 307 phospho-IRS-1 (Cell Signaling Technology #2381), total IRS-1 (Cell Signaling Technology #3407), Serine 256 phospho-FOX01 (ORIGENE #TA323072), total FOX01 (Cell Signaling Technology #2880), Serine 9 phospho-GSK3β (Cell Signaling Technology #9322), and total GSK3β (Cell Signaling Technology #9315), as previously described [60]. IRS-1 phosphorylation at Ser307 reduces IRS-1 activity; similarly, pSer256 can inhibit the function of FOXO1 and pSer9 reduces GSK-3β activity. Primary antibodies were diluted to a 1:2000 concentration and secondary antibodies were diluted 1:5000 concentration. Protein levels were quantified by image density scanning (LI-COR Odyssey). Protein levels are presented relative to β-actin (Abcam #ab20272) expression as described above. The number of biological samples used for western blotting and analysis is listed in each individual graph.

### 2.9 Statistical Analysis

Data were log-transformed as necessary. For statistical analyses, GraphPad Prism version 10.1 (GraphPad Software, Boston, MA) was used. A two-way repeated measures analysis of variance (independent factor: treatment; repeated measures factor: time) was used to examine growth, ITT, and GTT curves. We used GraphPad Prism (version 10.3.1) to estimate the area under the curve (AUC) as described in the online Prism 10 Statistics Guide (https://www.graphpad.com/guides/prism/latest/statistics/stat_area_under_the_curve.htm) for ITT and GTT. Given the known sex differences in response to high-fat diets, with male mice exhibiting significantly greater inflammation, insulin resistance, and obesity associated with high-fat diets compared to female mice [61-67], analyses were conducted separately for male and female mice. The groups (colchicine and placebo) were compared using t-tests (unpaired). Colchicine’s effects versus placebo on insulin sensitivity, body composition markers, systemic inflammation markers, and hepatic protein expression were assessed by unpaired two-tailed t-tests. The rejection of the null hypothesis was set at *p* < .05. We hypothesized that colchicine would improve insulin sensitivity by suppressing NLRP3-induced inflammatory mediators, without adversely affecting body composition.

## 3 RESULTS

### 3.1 Growth Curve and Body Composition

At the start of colchicine or placebo (after 4 weeks of high-fat diet), as anticipated, the male mice, who weighed 32.5 ± 2.9 g (mean ± SD), were significantly heavier that the female mice (22.6 ± 2.2 g, *p*<0.0001). Unexpectedly, growth curve analysis revealed time x treatment interaction effects, indicating that colchicine induced significantly less weight gain *vs.* placebo (Figs. **1B** and **1C**; *p*’s<0.0001). In male mice (Figs. **2A-F**), fat mass (Fig. **2A**; *p*=0.033) and fat percent (Fig. **2C**; *p*=0.005) decreased in colchicine *vs* placebo. We found no evidence to support a difference in lean mass (Fig. **2E**, *p* > 0.05). In the smaller cohort of female mice, there was no evidence to support differences in body composition outcomes (Figs. **2B**-**F**); however, there was marginal support for a decrease in total fat mass (Fig. **2B**; *p*=0.09) and fat mass percentage (Fig. **2D**; *p*=0.08) in colchicine versus placebo.

### 3.2 Insulin Tolerance Test

In males, ITT glucose concentrations were greater for colchicine *vs.* placebo from 60-120 min (all *p*< 0.05; Fig. **3A**). Colchicine increased ITT AUC glucose as compared to placebo (20,645 *vs* 18,254 mg/dL*min; *p* = 0.02; Fig. **3B**). In females, there was no evidence to support between-group differences of ITT glucose concentrations (Fig. **3C**) or ITT AUC values (Fig. **3D**).

### 3.3 Glucose Tolerance Test

In males, no glucose concentration differences between groups were found at any timepoint during the GTT (Fig. **3E**) or in GTT AUC glucose (Fig. **3F**). In females, during the GTT colchicine showed a group x time effect on glucose (Fig. **3G**; *p* = 0.006) and GTT glucose concentrations were less for colchicine *vs* placebo at 30 min (*p* = 0.026). However, there was no evidence to support differences in GTT AUC glucose for colchicine and placebo (Fig. **3H**; 27,114 *vs* 30,789 mg/dL*min; *p* = 0.13).

### 3.4 Inflammatory Markers

Serum CRP was decreased in the colchicine group *vs.* placebo, both for males (Fig. **4A**; 7.5 *vs.* 11.2 mcg/mL; *p* < 0.001) and females (Fig. **4B**; 5.9 *vs.* 7.9 mcg/mL; p = 0.026). We found no statistical evidence to support differences in hepatic NLRP3 (Figs. **4C** and **4D**) or cleaved (mature, ~22kD) Caspase-1 protein expression (Figs. **4E** and **4F**).

### 3.5 Skeletal Muscle Insulin Sensitivity

In male mice, expressed relative to B-Actin, there were no differences in skeletal muscle expression of pSer307 IRS-1 (pIRS-1), which reduces insulin action, pSer9 GSK-3B (pGSK-3 B), which negatively regulates its kinase activity that impedes glycogen synthesis in response to insulin, pSer256 FOXO1 (pFOXO1), which occurs in response to insulin activation of protein kinase B and inhibits FOXO1 function, or total IRS-1 (Figs. **5A**-**D**), but lower total GSK-3B (Fig. **5E**) and total FOXO1 (Fig. **5F**) were found for the colchicine group. Additionally, male mice administered IP colchicine showed a lower pIRS-1:total IRS-1 ratio compared to the placebo (Fig. **5G**; *p* < 0.05). Conversely, there was a greater pGSK3B:total GSK3B ratio (Fig. **5H**; *p* = 0.002) and pFOXO1:total FOXO1 ratio (Fig. **5I**; *p* = 0.0003) in male mice receiving colchicine. With female mice, there was no evidence to support differences between groups in any of these values except for a lower pFOXO1 expression in colchicine-treated mice (Figs. **6A**-**6I**).

In exploratory post-hoc analyses, including treatment, sex and the sex x treatment interaction in ANOVA models (ANOVA with repeated measures for body weight) did not alter the direction or significance of any finding for treatment and there were no significant treatment x sex interactions identified for any variable studied, though there were differences by sex. For example, for body weight, colchicine-treated mice weighed less than placebo-treated mice (*p*=0.11) and female mice were significantly lighter than male mice (*p*<0.001), but the treatment x sex interaction was not significant (*p*=0.130). For CRP, colchicine-treated mice had lower CRP than placebo mice (*p*=0.002) and female mice had lower CRP concentrations (*p*=0.005), but the treatment x sex interaction was not significant (*p*=0 307).

## 4 DISCUSSION

In this study of HFD mice, 4 weeks of IP colchicine injections at 0.2 mg/kg/d diminished body weight, adipose tissue growth, and serum CRP levels compared to placebo in both male and female mice, without significantly altering hepatic NLRP3 or Caspase-1 protein expression. We also observed reduced skeletal muscle pSer307 IRS-1, reduced total FOXO1, and increased pSer256 FOXO1 in male mice. Despite these findings, colchicine at 0.2 mg/kg worsened whole-body insulin resistance relative to the placebo in male mice, as evidenced by the ITT results, and in female mice, as evidenced by the high glucose levels during the GTT. Despite reduced pIRS-1 and increased pFOXO1 phosphorylation, suggesting improved insulin signaling, the high concentration of colchicine may have impaired vesicular transport mechanisms critical for GLUT4 translocation and overridden these benefits.

In this experiment, despite the abrogation of HFD-induced weight gain and CRP levels by colchicine, we were unable to demonstrate improvements in glucose homeostasis through modulation of NLRP3 inflammation activation by colchicine. We hypothesize that metabolic pathways, possibly related to pancreatic insulin secretion and/or peripheral insulin signal transduction, may have been negatively affected by the robust microtubular inhibition caused by the high dose of colchicine [68], thereby preventing us from observing any beneficial metabolic impact of inflammation reduction in these mice.

We observed that chronic injections of colchicine at 0.2 mg/kg did not reduce NLRP3 inflammasome protein expression in the liver when compared to placebo, with significant alterations in neither total NLRP3 nor cleaved (mature) caspase-1. Prior studies have found a very limited impact of colchicine injections on total NLRP3 expression in the AngII-induced mouse model [50], whereas they usually find that colchicine reduces cleaved caspase-1 expression or cleaved IL-1β expression [21, 23]. Since small to medium molecules, such as colchicine, are present in the peritoneal space, they are mostly absorbed *via* diffusion through mesenteric capillaries that feed the portal vein [69]. Therefore, it is expected that hepatic cells would be well-exposed to colchicine following intraperitoneal administration. Regardless of its initial entry route, most colchicine metabolism is hepatic [70]. In the present study, it appears likely that colchicine successfully prevented NLRP3 inflammasome assembly and its associated inflammatory consequences, as evidenced by a decrease in serum CRP concentration, even though it did not alter the proteins we monitored [71]. We expect that higher colchicine doses, which might have led to significant reductions in protein expression, would likely result in greater toxicity. Indeed, among both male and female mice, it was already evident that colchicine at 0.2 mg/kg prevented normal HFD-induced weight gain. For comparison, standard human colchicine doses are, on a per kg basis, approximately one-tenth of the doses used in the current mouse study (commonly used in other murine studies) and often lead to gastrointestinal side effects. At high doses, colchicine inhibits mitosis and has a greater impact on rapidly dividing cells, such as the gastrointestinal epithelium. This effect may potentially explain the observed weight loss [72].

Male mice demonstrate a more robust proinflammatory response to high-fat diet obesity compared to female mice [61, 63], so we expected to see a decrease in inflammation in response to colchicine primarily among male mice. Indeed, colchicine lowered pIRS-1 Ser307:IRS-1 and increased pFOXO1:FOXO1 in male mice, which should have significantly improved skeletal insulin sensitivity. However, as per the ITT, the glycemic parameters were substantially worse. These results suggest that colchicine at such high levels may damage the insulin transduction signal distal to FOXO1, resulting in peripheral insulin resistance. This is plausible because microtubules are essential to facilitate GLUT4 storage vesicle fusion to the plasma membrane [73]. Lower doses of colchicine may potentially avoid toxicity and yield improved metabolic outcomes.

## CONCLUSION

Recent reports on cytokine-specific pathways downstream of NLRP3 show favorable metabolic effects and demonstrate the need to focus on upstream components of the inflammatory cascade [15-17]. However, our data show no evidence for improved glucose metabolism in obese mice given 0.2 mg/kg colchicine. While colchicine effectively mitigated systemic inflammation and weight gain in HFD-fed mice, it did not confer metabolic improvements and may exacerbate insulin resistance at the tested dose. We conclude that investigators seeking to understand the impact of chronic colchicine exposure in mouse models should avoid high-dose IP colchicine dosing, as 0.2 mg/kg prevented normal HFD-related weight gain without improving insulin sensitivity.

## LIMITATIONS, STRENGTHS, AND FUTURE DIRECTIONS

This study is primarily limited by its focus on only one dose regimen and the use of a 45% high-fat diet, which may not have optimally increased the inflammatory milieu of the mice. Strengths of this study include employing a long exposure to colchicine to suppress obesity-linked inflammatory processes more proximally by inhibiting NLRP3 activation in mice with HFD-induced inflammation. Future studies could consider repeating the experiment using lower colchicine doses [74], possibly slowly increasing intraperitoneal dose to avoid toxicity [53], or using oral administration [75] and possibly studying a higher fat diet (*e.g.*, 60% kcal from fat diet instead of 45% HFD) to induce more consistent gains in body weight, adiposity, and systemic inflammation [76].

## Figures and Tables

**Fig. (1):**
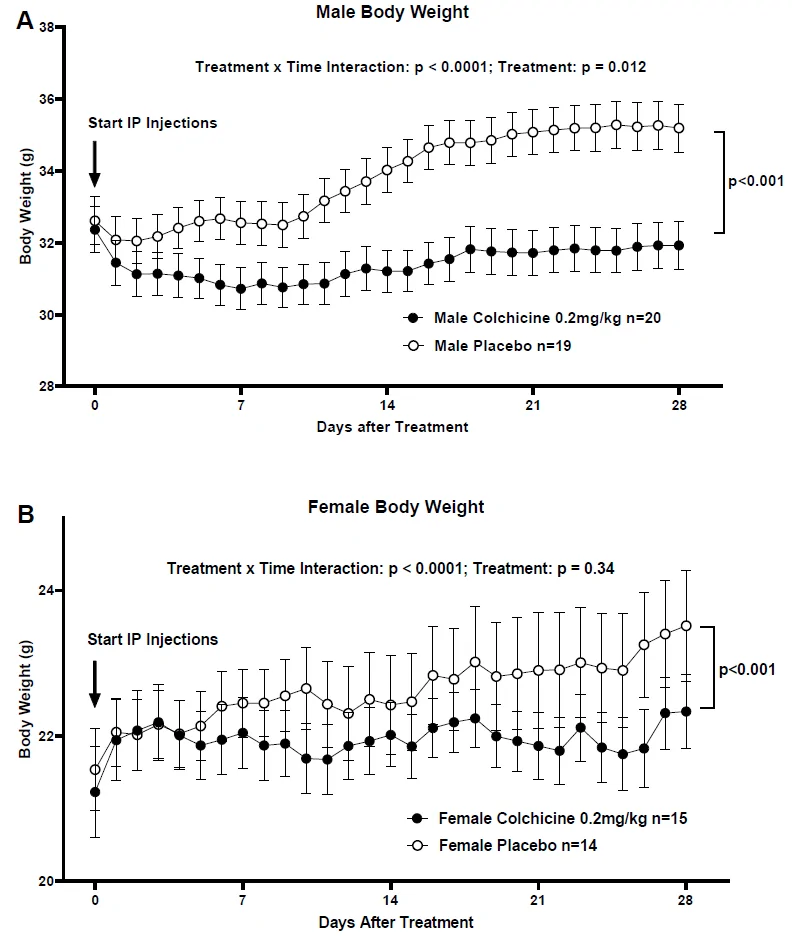
Effects of Colchicine on Body Weight. Body Weight trajectories for male (**A**) and female (**B**) mice treated with a high fat diet starting at 8 weeks of age, and daily intraperitoneal colchicine or placebo for 4 weeks starting at 12 weeks of age. Sample sizes for each analysis are given within each graph. Colchicine administration induced significantly less weight gain in male and female mice.

**Fig. (2):**
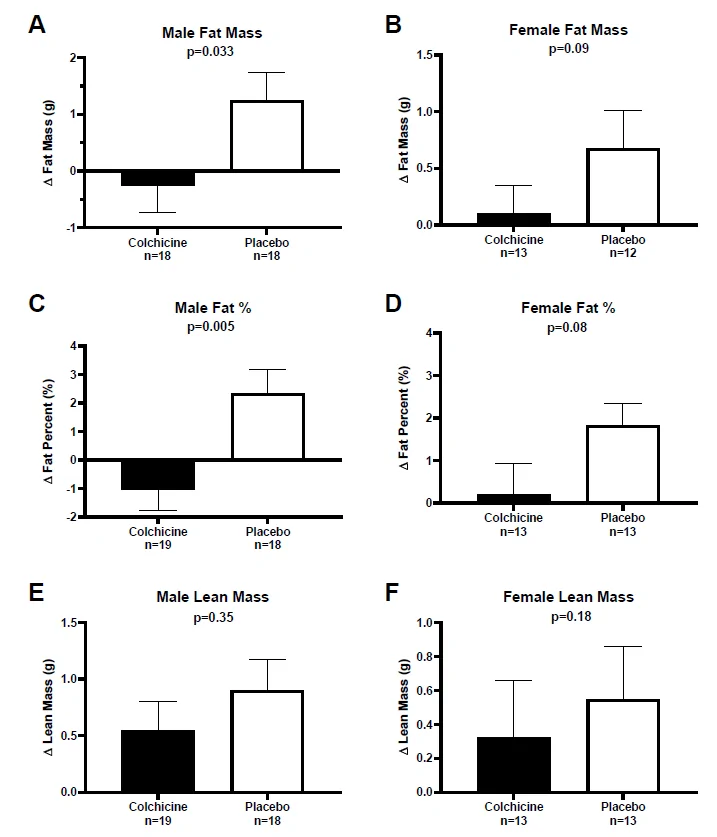
Effects of Colchicine on Body Composition. As determined by dual-energy x-ray absorptiometry, changes (∆) after 4 weeks of colchicine or placebo in fat mass (**A, B**), fat mass percentage (**C, D**), and lean mass (**E, F**) in male and female mice on a high-fat diet given colchicine (filled bars) or placebo (white bars). Sample sizes for each analysis are given within each graph. Fat mass and fat percent decreased significantly in colchicine *vs* placebo in male mice and marginally in female mice.

**Fig. (3):**
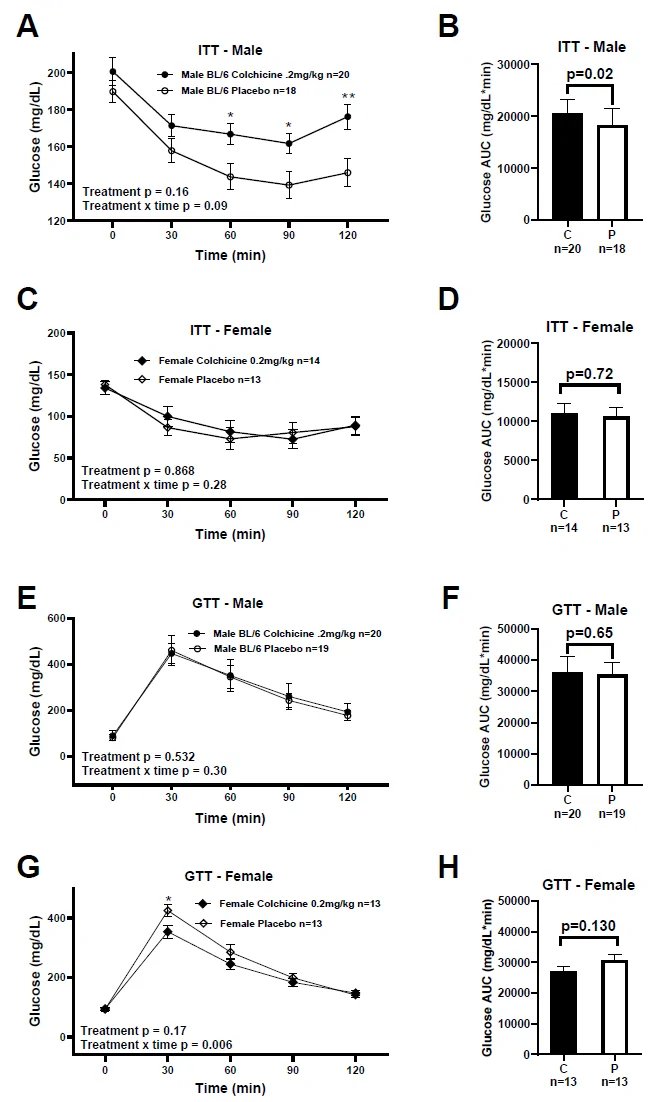
Effects of Colchicine on Glucose and Insulin Tolerance Tests. Insulin tolerance tests (ITT) performed in male (**A, B**) and female (**C, D**) mice. Glucose tolerance tests (GTT) performed in male (**E, F**) and female (**G, H**) mice. **Abbreviations:** AUC: Area under the Curve, calculated by the trapezoidal rule. C: colchicine; P: placebo. Sample sizes for each analysis are given within each graph. In males, ITT glucose concentrations were greater for colchicine *vs.* placebo from 60-120 min. In females, GTT glucose concentrations were less for colchicne *vs* placebo at 30 min.

**Fig. (4):**
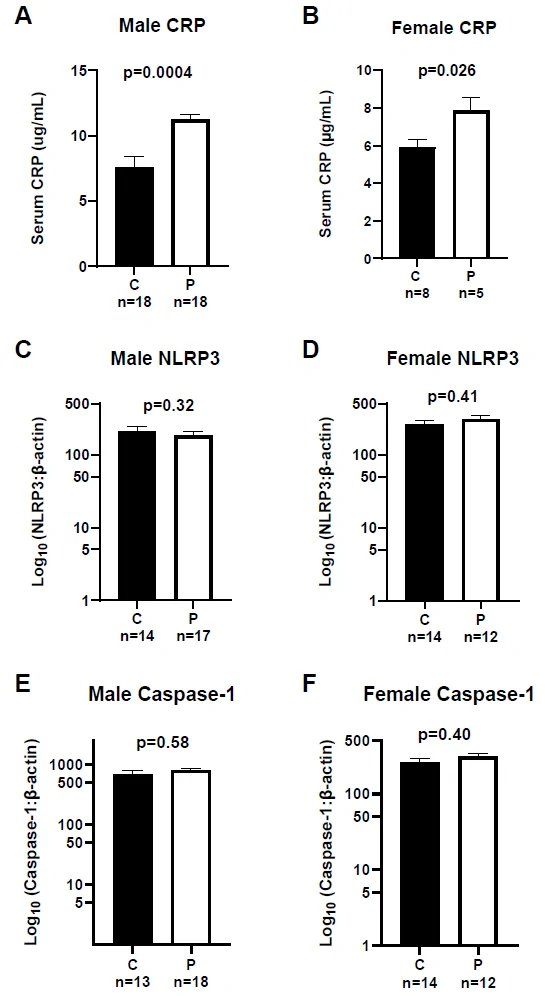
Effects of Colchicine on Inflammatory Biomarkers in Serum and Liver Samples. Serum C-Reactive Protein (CRP) in male (**A**) and female (**B**) mice. Results of Western Blot for hepatic NLRP3 (**C, D**) and cleaved (mature) Caspase-1 (**E, F**) in male and female mice. **Abbreviations:** C: colchicine; P: placebo. Sample sizes for each analysis are given within each graph. Serum CRP was decreased in male and female mice given colchicine *vs* placebo.

**Fig. (5):**
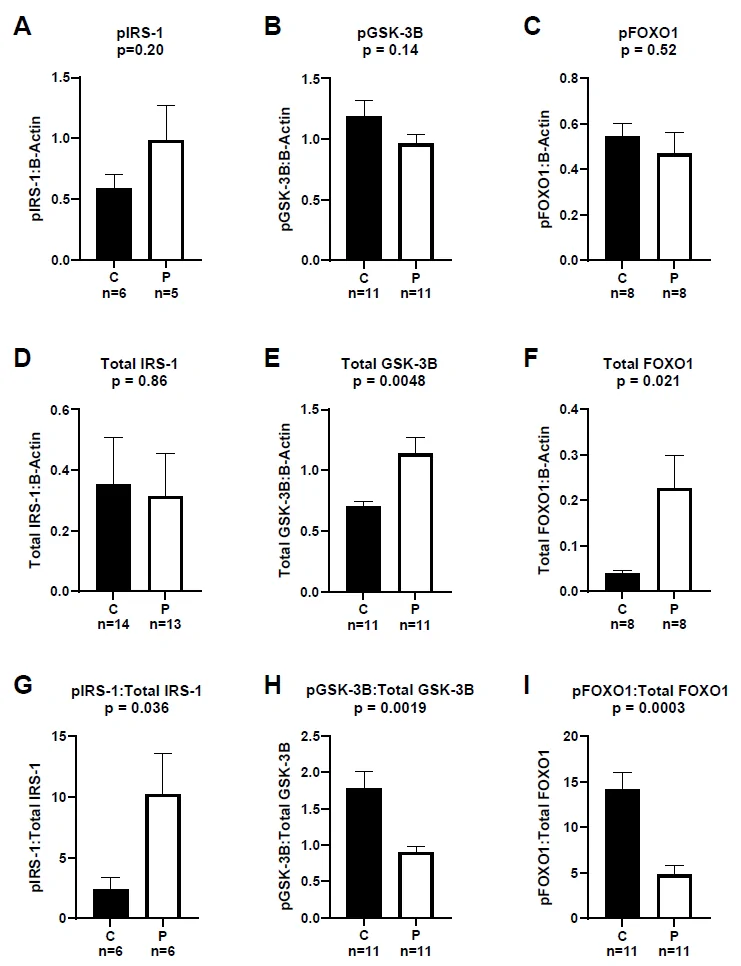
Effects of Colchicine on Male Skeletal Muscle. Western blots were performed on skeletal muscle samples for (**A**) IRS-1 phosphorylated at Ser307 (pIRS-1), (**B**) GSK-3B phosphorylated at Ser9 (pGSK-3B), (**C**) FOXO1 phosphorylated at Ser256 (pFOXO1), (**D**) total IRS-1, (**E**) total GSK-3B, (**F**) total FOXO1, and values calculated for ratios of (**G**) pIRS-1 to total IRS-1, (**H**) pGSK-3B to total GSK-3B, and (**I**) pFOXO1 to total FOXO1. **Abbreviations:** C: colchicine; P: placebo. Sample sizes for each analysis are given within each graph. Colchicine reduced skeletal muscle pSer307 IRS-1, total FOXO1, and increased pSer256 FOXO1 in male mice.

**Fig. (6):**
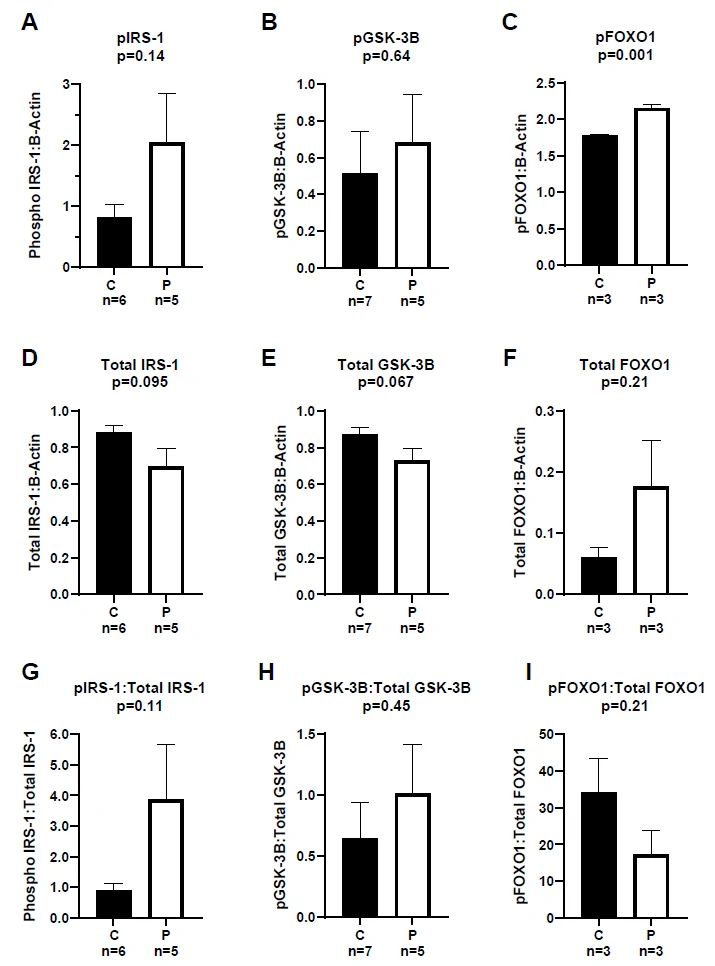
Effects of Colchicine on Female Skeletal Muscle. Western blots were performed on skeletal muscle samples for (**A**) IRS-1 phosphorylated at Ser307 (pIRS-1), (**B**) GSK-3B phosphorylated at Ser9 (pGSK-3B), (**C**) FOXO1 phosphorylated at Ser256 (pFOXO1), (**D**) total IRS1, (**E**) total GSK-3B, (**F**) total FOXO1, and values calculated for ratios of (**G**) pIRS-1 to total IRS-1, (**H**) pGSK-3B to total GSK-3B, and (**I**) pFOXO1 to total FOXO1. **Abbreviations:** C: colchicine; P: placebo. Sample sizes for each analysis are given within each graph. Colchicine reduced skeletal muscle pSer307 IRS-1, reduced total FOXO1, and increased pSer256 FOXO1 in male mice.

## Data Availability

The data for this manuscript are available at: https://data.mendeley.com/datasets/hmtgd7dnrb/1.

## LIST OF ABBREVIATIONS

IP: Intraperitoneal
HFD: High-Fat Diet
SOCS: Suppression of Cytokine Signaling
CRP: C-Reactive Protein
BW: Body Weight
